# Porous Copolymers of 3-(Trimethoxysilyl)propyl Methacrylate with Trimethylpropane Trimethacrylate Preparation: Structural Characterization and Thermal Degradation

**DOI:** 10.3390/ma17194796

**Published:** 2024-09-29

**Authors:** Małgorzata Maciejewska, Magdalena Rogulska

**Affiliations:** Department of Polymer Chemistry, Institute of Chemical Sciences, Faculty of Chemistry, Maria Curie-Skłodowska University in Lublin, Gliniana 33, 20-614 Lublin, Poland; mrogulska@umcs.pl

**Keywords:** porous microspheres, internal structure, thermal resistance

## Abstract

Porous polymeric microspheres are among the most effective adsorbents. They can be synthesized from numerous monomers using different kinds of polymerization techniques with a broad selection of synthesis factors. The main goal of this study was to prepare copolymeric microspheres and establish the relationship between copolymerization parameters and the porosity and thermal stability of the newly synthesized materials. Porous microspheres were obtained via heterogenous radical copolymerization using 3-(trimethoxysilyl)propyl methacrylate (TMPSM) as functional monomers and trimethylolpropane trimethacrylate (TRIM) as the crosslinker. In the course of the copolymerization, toluene or chlorobenzene was used as the pore-forming diluent. Consequently, highly porous microspheres were produced. Their specific surface area was established by a nitrogen adsorption/desorption method and it was in the range of 382 m^2^/g to 457 m^2^/g for toluene and 357–500 m^2^/g in the case of chlorobenzene. The thermal degradation process was monitored by thermogravimetry and differential scanning calorimetry methods in inert and oxidative conditions. The copolymers were stable up to 269–283 °C in a helium atmosphere, whereas in synthetic air the range was 266–298 °C, as determined by the temperature of 5% mass loss. Thermal stability of the investigated copolymers increased along with an increasing TMPSM amount in the copolymerization mixture. In addition, the poly(TMSPM-*co*-TRIM) copolymers were effectively used as the stationary phase in GC analyses.

## 1. Introduction

Material porosity is a property that gives the material its unique characteristics. Guest molecules can be selectively adsorbed into the regions of empty space in porous materials. Accordingly, they are extensively exploited as adsorbents in different types of chromatography [1,2,3,4], heavy metal sorption or water contamination removal [5,6,7,8,9,10,11,12]. Pores may also serve as reservoirs for the controlled release of formerly absorbed substances like in drug delivery systems [13,14,15]. They may also adsorb catalytically active species or exhibit catalytic properties themselves. Consequently, the host molecule can be even chemically transformed inside them. There are several different types of porous material including, but not limited to, activated carbons, zeolites, silicas, metal organic frameworks (MOFs), porous organic polymers (POPs), and microporous organic polymers(MOPs). The size and chemical composition of the pores determine the direction of their usage. They might be utilized for the selective adsorption or storage of required molecules.

POPs are composed predominantly of light, non-metallic elements such as carbon, oxygen, hydrogen, nitrogen, and boron. The majority of POPs are chemically stable under various environmental conditions. Additionally, the organic nature of POPs allows for convenient modification. The functional groups present in many polymer backbones can be modified, providing POPs with tailored properties for specific applications [16,17,18,19,20,21].

MOPs are a specific subset of porous organic polymers characterized by their microporosity. They have very high specific surface areas and pores with diameters not exceeding 2 nm [22]. The very high physical surface area exhibited by microporous materials (usually 300–3000 m/g, as measured by well-known techniques like the Brunauer, Emmet, and Teller (BET) models [23]) is the key to their many applications Microporous materials hold significant positions in numerous fields, including but not limited to catalysis and separation science. The pore dimensions are comparable to small molecules. The microporous structure allows for the selective adsorption of small molecules and gases. Many MOPs demonstrate good thermal and chemical stability, making them suitable for various industrial and environmental implementations. Because of their high surface area and tunable properties, MOPs can serve as efficient catalysts or supports for catalytic processes [24,25,26].

Although the class of highly porous polymers can be considered as quite mature, the need for improved materials remains urgent. The response to this problem can be provided by incorporation into the polymeric matrix 3-(trimethoxysilyl)propyl methacrylate(TMSPM). It contains tri-alkoxy groups and can be supportive in adhesion promotion between dissimilar materials. The Si-O-CH_3_ can work as the bonding agent between polymers and metals. Consequently, TMSPM was successfully used, e.g., in dentistry. The composite resin’s mechanical properties and durability increased with a silanized filler [27].

TMPSM was successfully applied as a three dimensional printed porous methacrylate/silica hybrid scaffold for bone replacement [28] as well as for various surface modifications of filler particles to obtain the best compatibility of the filler with the polymeric phase [29].

As to other fields, TMPSM was effectively applied in aluminum–vegetable oil composite [30] and successfully attached to natural rubber particles in a colloid state [31]. Also, metal-to-rubber bonding by this silane coupling agent [32] as well as performance improvement of alumina/silicone rubber composites [33] were investigated. It was also presented that using alkoxysilanes as a modifier can significantly improve the adhesion between the filler and the polymeric matrix filler [34]. In the work of Prosvirnina et al. [35], cellulose nanofibers(CNF) made by bacteria were modified with TMSPM and used as a reinforcing and crosslinking agent for inks that are 3D printable and UV-curable. The application of TMSPM resulted in an improvement of 3D printed filaments quality because of diminished CNF agglomeration.

TMSPM was also successfully applied to miscellaneous inorganic–organic hybrid materials, e.g., poly(acrylic acid)-[3-(trimethoxysilyl)propyl methacrylate]-Al_2_O_3_ [36] and vinyl chloride/3-(trimethoxysilyl) propylmethacrylate-intercalated Mg-Al-hydroxide layered double hydroxide [37]. An intriguing hybrid material based on 3-(trimethoxysilyl)propyl methacrylate and octafunctionalized cage-like polyhedral oligomeric silsesquioxane for bone replacing was presented by John et al. [38].

Furthermore, TMSPM can be found in nanoemulsions [39]; an amphiphilic, pH-sensitive hydrogel network [40]; thermoresponsive hybrid micelles consisting of a cross-linked thermoresponsive hybrid hydrophilic shell and a hydrophobic core domain [41]; and xerogels [42]. An interesting case is a drug-release system based on thermoresponsive sol–gel hybrid hydrogels of poly(*N*-isopropylacrylamide-*co*-3-(trimethoxysilyl)propyl methacrylate) copolymers. This system contains a relatively high TMSPM amount and is theophylline-loaded. What is interesting is that the gels released theophylline not only below but also above the gel collapse temperature, with a significant amount of the drug [43].

Tham et al. investigated the effect of TMSPM on the morphological, thermal, and other properties of hydroxyapatite/poly(methyl methacrylate) composites [44]. It was found that *T*_10_ (the onset temperature of the degradation at which 10% weight loss occurred) of the composites increased from 246 °C to 270 °C along with an increase of TMSPM content from 2% to 8%. At the same time, *T*_50_ (the midpoint temperature at which 50% weight loss took place) rose from 334 °C to 354 °C. This phenomenon may be explained by the improvement of interfacial bonding between the functionalized filler and the polymeric network. It can generate a barrier effect on the volatile molecules produced during the thermal degradation of the poly(methyl methacrylate) matrix.

The presented study is devoted to the synthesis of permanently porous copolymers of TMPSM crosslinked with trimethylolpropane trimethacrylate (TRIM) in the form of regular microspheres. The main aim of this research was to establish the correlation between polymerization parameters, especially different concentration of TMPSM, on structural and thermal properties of the newly synthesized copolymers. For this purpose, a low-temperature nitrogen adsorption/desorption method, ATR-FTIR spectroscopy, scanning electron microscopy differential scanning calorimetry and thermogravimetry were applied. Additionally, GC analyses were conducted.

## 2. Materials and Methods

### 2.1. Chemicals

TMSPM from Acros Organics (Geel, Belgium) and TRIM from Sigma–Aldrich (Schnelldorf, Germany) were used as received. Bis(2-ethylhexyl) sulfosuccinate sodium salt (AOT) and 2,2′-azoisobutyronitrile (AIBN) from Fluka AG (Buchs, Switzerland) were also applied without purification. Acetone, chlorobenzene, toluene, benzene, pentan-2-one, methanol, butan-1-ol, and sodium hydroxide (reagent grade) were from POCh (Gliwice, Poland).

### 2.2. Synthesis of Permanently Porous Microspheres

**Figure 1:**
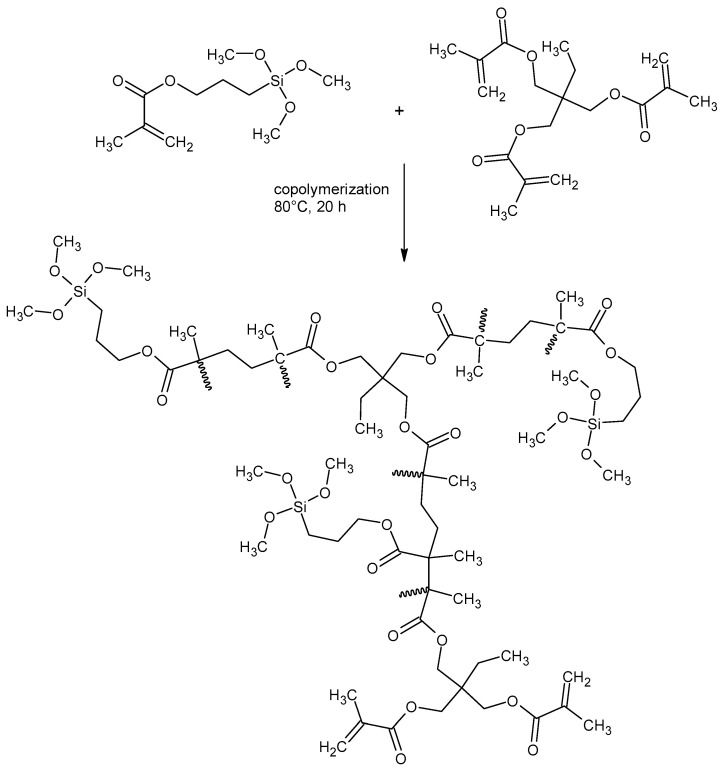
Illustrative scheme of the copolymerization of TMSPM with TRIM.

In the first step, the continuous phase was prepared by dissolving 2.2 g of a surfactant (AOT) in 195 mL of deionized water. Then, a solution of 15 g of monomers (TMSPM and TRIM), and 0.2 g of an initiator (AIBN) in 22.5 mL of pore-forming agent (toluene or chlorobenzene) was prepared and added to the aqueous medium without stopping the stirring. Molar ratios of TMSPM to TRIM were changed from 1:1 to 4:1. The experimental data of the syntheses are collected in Table 1. Copolymerization was conducted for 20 h at 80 °C and the obtained porous beads were filtered off using 5 μm filter paper. Next, an intensive cleaning process was implemented in order to remove the unreacted monomers, diluent, and physically adsorbed surfactant. The microspheres were put into a beaker, dispersed in deionized water and sonicated for 0.5 h in an ultrasonic bath. After removing the water, the microspheres were resuspended in methanol and sonicated for 0.5 h. In the next step, the methanol was discarded and the microspheres were transferred into a round-bottomed flask, mixed with toluene, and stirred for 0.5 h. At that point, the toluene was removed and the microspheres were stirred with methanol for 0.5 h. The methanol was infiltrated, and the microspheres were washed with deionized water, filtered, and dried in a vacuum oven at 60 °C for 48 h. Figure 1 presents the general route of the synthesis.

### 2.3. Measurement Methods

Fourier transform infrared (FTIR) spectra were recorded on a Tensor 27 FTIR spectrometer (Bruker Corp., Ettlingen, Germany) using an attenuated total reflectance (ATR) technique. They were collected in the 4000–600 cm^−1^ spectral range with a resolution of 4 cm^−1^. For each spectrum, 32 scans were completed.

An ASAP 2405N analyzer (Micromeritics Corp., Norcross, GA, USA) was used for the determination of nitrogen adsorption/desorption isotherms at −196 °C. Before the measurements, the samples underwent outgassing under vacuum (10^−2^ mm Hg) overnight at 80 °C. The Brunauer–Emmet–Teller (BET) approach was applied to estimate the specific surface area (S_BET_), whereas the Barrett, Joyner and Halenda (BJH) approach was used for the determination of pore volume (V), pore size distributions (PSD) and its maxima (PSD_max_). The pore diameters (D_BJH_) were estimated in accordance with the PSD_max_.

Scanning electron microscope (SEM) Duall BeamTM, Quanta3D FEG (Fei Corp., Hillsboro, OR, USA) working at 5 kV was applied for taking the images of the synthesized microspheres. Before measurements, they were coated with a thin layer of gold.

A DSC 204 apparatus (Netzsch, Selb, Germany) operating in a dynamic mode was employed for calorimetric measurements. The measurements were conducted from room temperature to 500 °C in aluminum crucibles with pierced lids (mass of about 40  mg). As a reference, an empty aluminum crucible was used. A dry inert gas (argon) with a flow rate of 30 mL/min was flushed through the cells. Samples of 10 ± 0.2 mg were used. The heating rate was kept at 10 °C/min.

The size distribution of the synthesized microspheres was determined using a Mastersized Analyser 2000 (Malvern, Instruments Ltd., Worcestershire, UK). The statistical data of the distribution were obtained on the basis of the derived diameters according to the British standard BS2955:1993. In accordance with this standard, D(0.1) is the size in microns of particles below which 10% of the sample lies and D(0.9) is the size of particles below which 90% of the sample lies. D(0.5) refers to Mass Median Diameters (MMD), that is, the size in microns at which 50% of the sample is smaller and 50% is larger. Span (width of the size distribution) was calculated as:

$$span=\frac{d(0.9)-d(0.1)}{d(0.5)}$$

A Netzsch STA 449 F1 Jupiter thermal analyzer (Netzsch, Selb, Germany) was used for the TG analyses. They started at 35 °C and ended at 700 °C. The measurements were conducted in helium or synthetic air (flow = 20 mL/min),. The heating rate was equal 10 °C/min, whereas the sample mass was about 10  mg. To remove the absorbed water and thus obtain reliable information on the decomposition of the copolymers, the samples were heated at 100 °C before the analyses. All of the measurements were conducted in Al_2_O_3_ pans (mass of about 160 mg). As a reference, an empty Al_2_O_3_ pan was applied. The volatile products emitted during the heating of the selected copolymeric microspheres were analyzed by a Bruker Tensor 27 FTIR spectrometer (Bruker Corporation, Hanau, Germany) coupled online to a Netzsch STA instrument. The spectrometer was connected by a Teflon transfer line (2 mm diameter) heated to 200 °C to avoid the condensation of volatiles. The FTIR spectra of the gaseous products were collected in the range of 600–4000 cm^−1^ with sixteen scans per spectrum. The required measurement resolution was 4 cm^−1^.

Chromatographic measurements were carried out on a gas chromatograph GC 1000 (Dani, Milan, Italy) supplied with an injector (220 °C) and a thermal conductivity detector (TCD, 220 °C). As a carrier gas, helium, at a flow rate of 50 mL/min, was used. The samples were injected manually using a syringe of 1 μL volume (SGE, North Melbourne, Aus-tralia). All measurements were conducted at 200 °C.

The swellability coefficient, *B*, was determined by equilibrium swelling in acetone, methanol, and toluene using the centrifugation method. *B* was calculated as:

$$B=\frac{V_{s}-V_{d}}{V_{s}}\times 100\%$$

where *V_s_* is the volume of the copolymer after swelling and *V_d_* is the volume of the dry copolymer.

In addition, the swelling process was controlled using a G3 (Malvern, Instruments Ltd., Worcestershire, UK).

## 3. Results and Discussion

**Figure 2:**
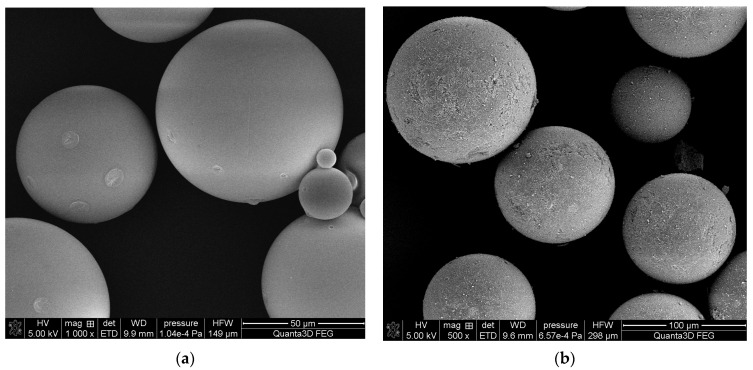
SEM images of (**a**) TMSPM-*co*-TRIM_1T copolymer; (**b**) TMSPM-*co*-TRIM_1C copolymer.

Porous microspheres of the TMSPM cross-linked with TRIM were obtained via heterogenous radical copolymerization. In their synthesis, a modified suspension technique was applied. The typically used high molecular mass stabilizer was replaced by a surfactant (AOT). As the concentration of the surfactant in the water medium exceeded its critical micelle concentration, it was present in the form of micelles. The polymerization process took place in the micelles. Thanks to this, copolymeric microspheres in the diameter range of 10–180 µm were obtained (Figure 2).

**Figure 3:**
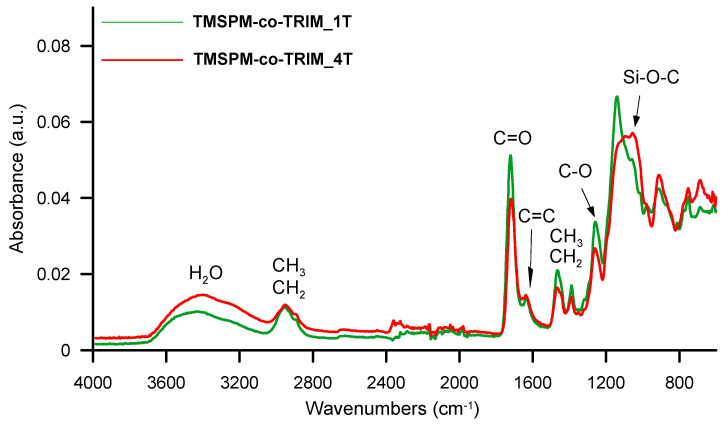
ATR-FTIR spectra of selected copolymeric microspheres.

In order to endorse the assumed chemical structure of the synthesized copolymers, an ATR-FTIR method was used. The polymeric network is mainly composed of units derived from methacrylate groups. Their presence is confirmed by the bands at 1721 cm^−1^ attributed to the C=O stretching vibrations as well as at 1259 cm^−1^ connected with the C–O stretching vibration. The incorporation of TMSPM into the polymeric matrix is evidenced by the band at 1056–1055 cm^−1^ associated with the asymmetrical Si–O–C stretching vibrations. Its intensity increased along with the increase in the amount of TMSPM in the polymerization mixture. The existence of methyl and methylene groups is indicated by the bands at ~2936–2870 cm^−1^ (C–H stretching vibrations) and at 1446 and 1388 cm^−1^ (C–H bending vibrations). A very low intense band at 1638–1631 cm^−1^ coming from the unreacted C=C double bonds is also visible. A broad band with a maximum at about 3400 cm^−1^ is associated with the adsorption of water molecules on the surface of the pore channels. Figure 3 presents the spectra obtained for the chosen copolymeric microspheres.

The main weakness of the free-radical heterogenous polymerization is a broad distribution of the microsphere diameters. Their average size depends on many parameters, including the diameter of the stirrer, the diameter of the flask, the ratio of the organic phase to the water phase (*v*/*v*), the speed of mixing, and the surfactant concentration. Keeping the above-mentioned factors at the same level, differences in the microsphere diameters were still visible. This was the result of the various monomers ratios and different porogenic solvents. These factors were responsible for diverse densities in the organic phase. As a consequence, microspheres of various diameters were synthesized (Table 2).

The mass median diameter of the microspheres was in the 75–155 µm range. What is important, smaller beads were synthesized in the presence of toluene in the polymerization mixture. These copolymers also have a broader sized distribution and consequently, a higher value of spam. The copolymers obtained with chlorobenzene as the porogenic solvent were more uniform. The main aim of this research was to create new polymeric materials with a highly developed internal structure and a considerable amount of methoxysilyl units in the form of regular microspheres. To achieve this goal, different monomer concentrations (molar ratio from 1:1 to 1:4) and two diverse porogens were used. As it follows from the data collected in Table 3, all of the obtained copolymers are characterized by a high value of specific surface area. What is important, its value is not correlated with the particle size distribution.

In the case of copolymers obtained in the presence of toluene, the specific surface area increases along with the increment of crosslinking monomer (TRIM). This phenomenon is connected with the process of phase separation during the formation of the internal structure of the copolymers. The process of phase separation can be attributed to either an increase in the cross-linking degree (ν-syneresis) or a modification in the polymer–solvent interaction (macro- or microsyneresis). Due to a higher concentration of crosslinker in the polymerization mixture, early phase separation takes place and a huge number of low energy and highly crosslinked nuclei is created. Additionally, the adsorption of the remaining monomers and swelling of the newly formed nuclei is limited by their high crosslinking degree. After the nuclei aggregation, the resulting microgels are extremely complex and generate a highly developed internal structure.

**Figure 4:**
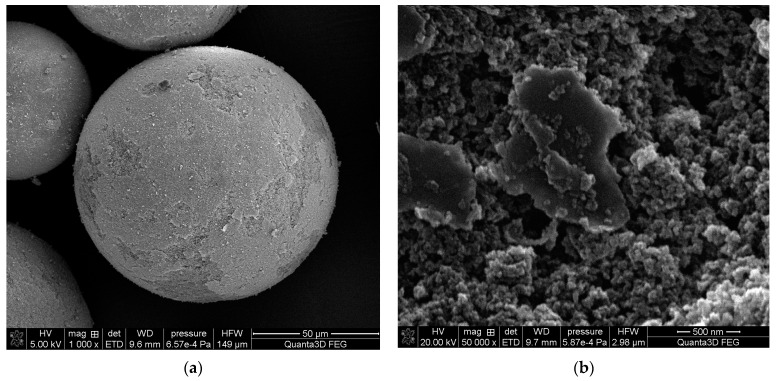
SEM images of TMSPM-*co*-TRIM_4C copolymer (**a**) zoom 1000×; (**b**) zoom 50,000×.

For copolymers synthesized with chlorobenzene as the pore-forming agent, the specific surface area expands with the increase in the amount of functional monomer. This trend is connected to the solvating power of the diluent. The Hildebrand solubility parameter of chlorobenzene is equal to 19.6 (MPa)^0.5^, whereas that of toluene is 18.2 (MPa)^0.5^. This difference is crucial because the phase separation strongly depends on the thermodynamic quality of the porogens. While the thermodynamic quality of the solvent increases, a good solvent can compete with monomers in the solvation process of nuclei. The monomer concentration in the nuclei is considerably lower and, consequently, the globules grow slower and remain miniature. For that reason, copolymers synthesized in thermodynamically good solvents have smaller pores and a higher specific surface area. The highly developed internal structure is depicted in the SEM images (Figure 4).

**Figure 5:**
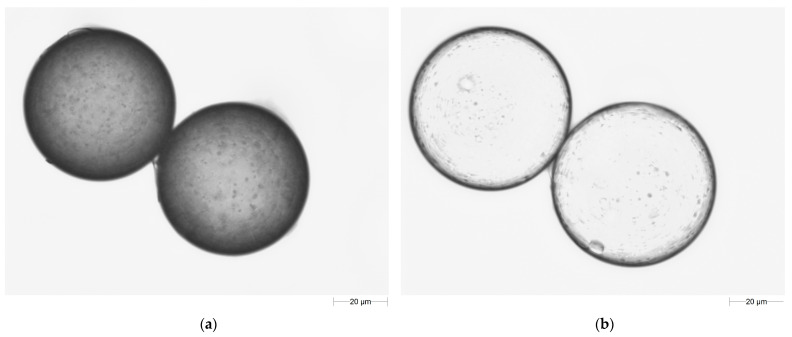
Microscope images of the TMSPM-*co*-TRIM_1T copolymer before (**a**) and after swelling in acetone (**b**).

The newly synthesized crosslinked porous microspheres possess the ability to swell. During the swelling, the pores are rapidly filled with the solvent. At the same time, the network takes up solvent from the environment. The extent of swelling depends on the total volume of pores and the attractive force between the network segments and solvent molecules. Figure 5 displays a magnified image of the TMSPM-*co*-TRIM_1T copolymer before and after swelling in acetone. For all of the studied copolymers, their swellability coefficients in methanol, toluene and acetone were determined These values are presented in Table 4.

As can be seen, the highest value of the coefficient is observed for copolymers with the most developed internal structure and highest amount of TMSPM in the polymeric network. The ability to swell is determined by the cross-linking density and interactions between solvent molecules and network chain. These two parameters are balanced in the case of copolymers synthesized with toluene as porogenic solvents. Consequently, their swellability coefficients are quite similar.

Thermal degradation of the copolymeric microspheres was studied in inert and oxidizing atmospheres. Because the preliminary investigations showed no difference in the thermal behavior of porous materials obtained with different porogens, herein we present the results for materials synthesized with the usage of toluene. Table 5 and Table 6 contain the basic parameters evaluated on the basis of TG and differential TG (DTG) curves. The initial decomposition temperature was defined as the temperature of 5% mass loss (*T*_5%_). In helium (see Table 5), the microspheres were stable up to 269–283 °C. Generally, in the polymerization of a bifunctional monomer (e.g., styrene, glycidyl methacrylate (GMA), *N*-vinyl-2-pyrrolidone, and hydroxyethylmetacrylate (HEMA)) with a multifunctional crosslinker (e.g., divinylbenzene, TRIM, 9,10-bis(methacryloyloxymethyl)anthracene, ethylene glycol dimethacrylate (EGDMA)) the thermal stability of the obtained copolymers increased with the crosslinker concentration [20,45,46,47,48]. In the investigated materials, the trend is the opposite. The stability increased along with the increase in the TMSPM amount. This phenomenon can be explained by the chemical structure of the functional monomer. The presence of the silyl species allows for the creation of varied three-dimensional forms. These rigid frameworks contribute to the reinforcement of the thermal resistance of the synthesized materials [49]. The mentioned *T*_5%_ values (269–283 °C) indicate that the newly obtained copolymers were characterized by similar or even better thermal stability compared to other porous microspheres. Material based on poly(GMA-*co*-TRIM) was stable to 238 °C, and that based on benzene-1,4-diylbis(2-methylprop-2-enoate) to 254 °C [50]. In turn, poly(HEMA-*co*-EGDMA) copolymers were thermally stable to about 250 °C [45], whereas poly(HEMA-*co*-TRIM) copolymers were stable to 251–281 °C depending on the molar ratio of the monomers [51]. The same relationship was observed for the temperatures of 20% mass loss (*T*_20%_) and 50% mass loss (*T*_50%_). They were in the ranges of 303–356 °C and 380–443 °C, respectively. In the synthetic air (see Table 6), the values of the characteristic temperature indicators were lower, except for *T*_5%_ determined for TMSPM-*co*-TRIM_3 and TMSPM-*co*-TRIM_4 copolymers with higher content silyl-based units. This phenomenon can be associated with the formation of more stable intermediates in the oxidizing atmosphere compared to the inert one. It is worth noticing that copolymers with a high amount of TMSPM (TMSPM-*co*-TRIM_3 and TMSPM-*co*-TRIM_4) showed better thermal stability than pure poly(TRIM) in both atmospheres [52].

**Figure 6:**
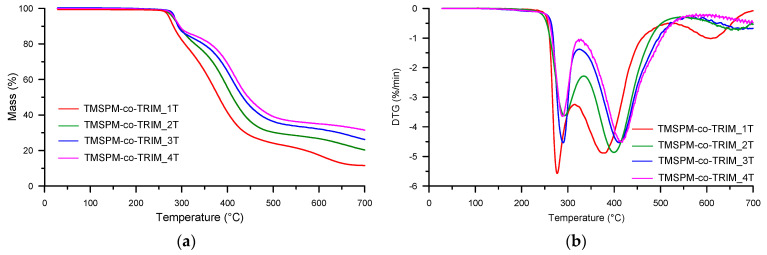
TG (**a**) and DTG (**b**) curves of the investigated copolymers determined in helium.

**Figure 7:**
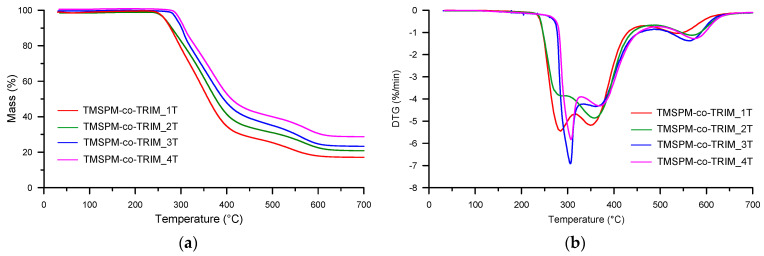
TG (**a**) and DTG (**b**) curves of the investigated copolymers determined in air.

The course of the TG-DTG curves (Figure 6 and Figure 7) indicated the multi-stage decomposition of all newly obtained copolymeric microspheres in both helium and synthetic air. Namely, in helium, on the DTG curves, three partly overlapping peaks were visible with the maxima in the range of 277–293 °C (assigned as *T*_max1_), 379–418 °C (assigned as *T*_max2_) and 608–712 °C (assigned as *T*_max3_). In turn, in the air atmosphere, the temperatures ranges were as follows: *T*_max1_: 286–307 °C, *T*_max2_: 349–369 °C and *T*_max3_: 537–574 °C. Even though these peaks are not completely separated, it is possible to approximately determine the mass losses corresponding to the given decomposition stages. The main mass loss (~40–55%) occurred in the second stage. It should be stressed that the residue was correlated with the molar ratio of the comonomers. The higher the content of TMSPM in the polymerization mixture, the higher the residual mass.

**Figure 8:**
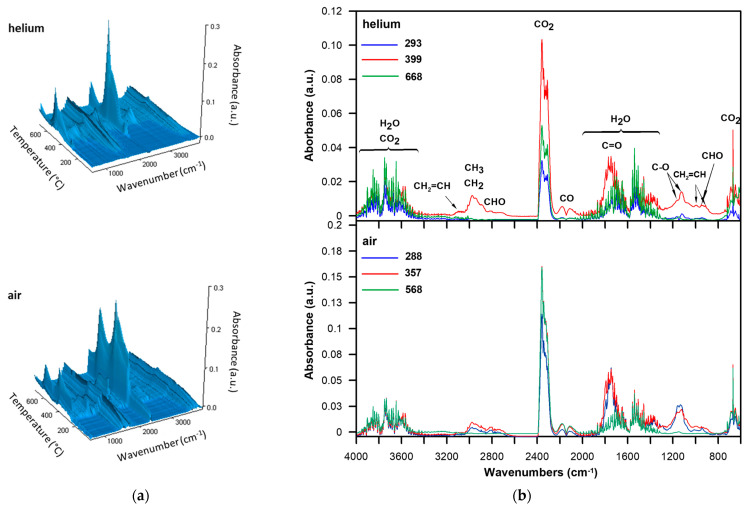
FTIR spectra of volatile products evolving during the thermal degradation of TMSPM-co-TRIM_2T: (**a**) in the whole temperature range of the measurement and (**b**) extracted at the maxima of degradation.

As can be seen in the FTIR spectra of volatiles emitted during the thermal degradation of TMSPM-*co*-TRIM_2T in helium (Figure 8), most of the compounds were formed in the second stage. The spectrum from the second maximum of degradation showed the absorption bands 1772 cm^−1^ characteristic of carbonyl organic products such as aldehydes and esters. The presence of aldehydes was demonstrated by the bands at 2821 and 2730 cm^−1^, associated with the C–H stretching vibrations and at 949 cm^−1^, related to the C–H deformation vibrations, while the bands at 1165–1124 cm^−1^, typical of the C–O stretching vibrations, were signs of the occurrence of esters. The spectrum also exhibited bands pointing to the formation of vinyl compounds (at 3099 cm^−1^ as well as at 912 and 989 cm^−1^, attributed to the C–H stretching and out-of-plane deformation vibrations, respectively) and aliphatic ones (at 2974 and 2866 cm^−1^, related to the asymmetric and symmetric C–H stretching vibrations, respectively, of the methylene and methyl groups). Moreover, bands typical of water (at about 4000–3500 cm^−1^, associated with the stretching vibrations and at about 1800–1300 cm^−1^ with the deformation vibrations), carbon dioxide (at 2359–2310 cm^−1^, ascribed to the asymmetric stretching vibrations, and at 669 cm^−1^ to the degenerate deformation vibrations) and carbon monoxide (at 2181 and 2114 cm^−1^, connected with the stretching vibrations) were visible. The spectra from the first and third maxima of degradation indicated the creation of mainly carbon dioxide, carbon monoxide, and water.

As for the degradation of this copolymer in an oxidative atmosphere (Figure 8), the release of gases of the same types as in the inert atmosphere was observed. The differences were visible in the amount of products formed. In the first and second stages, the amount of carbonyl, aliphatic and vinyl compounds was at a similar level, which indicates that the decomposition of methacrylate units occurred in both stages. The third stage is the emission of oxidation products of compounds created in the previous stages of degradation.

**Figure 9:**
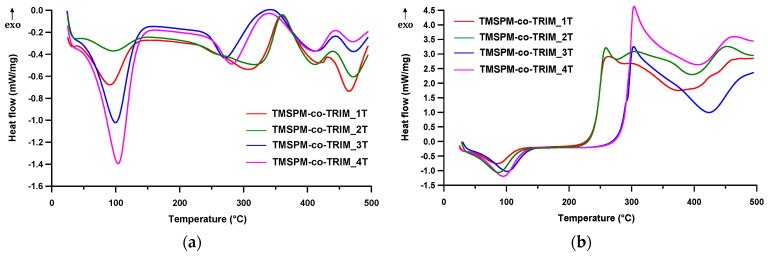
DSC curves of the investigated copolymers determined in helium (**a**) and air (**b**).

The thermal properties of the newly synthesized microspheres were also examined during DSC measurements conducted in two different atmospheres. Figure 9a displays the DSC curves of the investigated copolymers obtained in helium. As can be seen, both exothermic as well as endothermic thermal events took place. The first endothermic peak (the maximum at about 100 °C) is correlated with the moisture vaporization from the copolymers.

With the increase in the quantity of functional monomer (TMSPM) in the copolymer, the volume of adsorbed water considerably increases. There is only one exception to this rule: the observed effect for TMSPM-*co*-TRIM_1T is bigger compared with the one for TMSPM-*co*-TRIM_2T. This phenomenon can be explained by the more developed internal structure of TMSPM-*co*-TRIM_1T (especially in terms of pore volume) and its consequent higher adsorption capacity. The enthalpy of the evaporation process increased from 33 J/g for TMSPM-*co*-TRIM_2T to 299 J/g for the TMSPM-*co*-TRIM_4T copolymer. The second endothermic peak, whose maximum increased with the degree of crosslinking (from 271 to 316 °C), can be referred to as the degradation reactions of the ester groups. Its enthalpy is in the range 66–85 J/g depending on the monomer composition. The exothermic peak following the first step of endothermic decomposition can be attributed to a thermal crosslinking reaction of double bounds. They were formed in the first step of the thermal degradation by the β-bond scission reactions. The last endothermic peak is connected to the thermal degradation of the rigidly crosslinked part of the copolymers. The enthalpy of this process increases along with the increment of crosslinking degree from 18 J/g for TMSPM-*co*-TRIM_4T to 70 J/g for TMSPM-*co*-TRIM_1T. As regards the experiments conducted in air, mainly the exothermic peaks are visible on the DSC curves (Figure 9b). They considerably prevail over the minute endothermic peak at about 100 °C. An analogous one was also observed in the helium atmosphere. The exothermic peaks are connected to the degradation of the investigated polymers that occur via oxidation processes.

**Figure 10:**
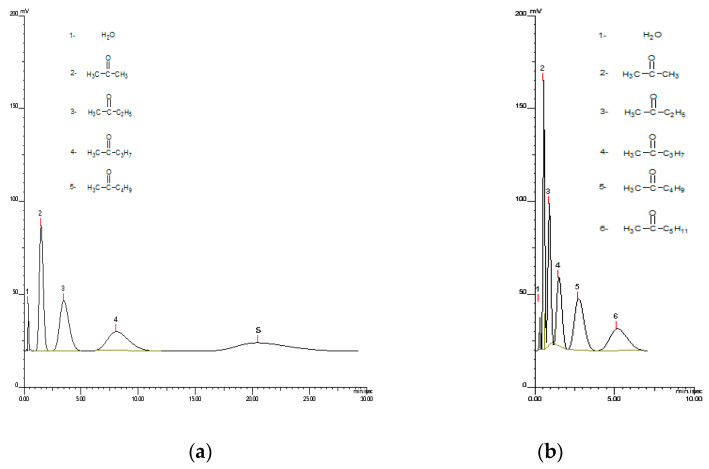
GC chromatograms of ketones separation at 140 °C (**a**) and 200 °C (**b**) conducted on poly(TMSPM-*co*-TRIM)_4T copolymer.

The high thermal stability of the obtained copolymers opened the possibility of their application as stationary phases in gas chromatography. Additionally, the presence of polar units offers hydrophilic properties. It permits the interaction of the poly(TMSPM-*co*-TRIM) copolymer with polar compounds. Figure 10 displays a chromatograph of a mixture of aliphatic ketones. The measurements were conducted at 140 °C and 200 °C. The time required for ketones separation was shorter at a higher temperature. What is more, at 200 °C, the peaks were not diffused. Thus, it can be assumed that copolymers with greater thermal stability are more useful for GC applications.

## 4. Conclusions

The presented research describes the synthesis and characterization of porous, thermally stable polymeric microspheres based on 3-(trimethoxysilyl)propyl methacrylate (TMPSM) and trimethylpropane trimethacrylate (TRIM). A modified suspension–emulsion polymerization was applied. The main goal of this study was to establish the correlation between polymerization parameters and the porosity and thermal stability of the newly synthesized materials. It was found that their structural properties were strongly correlated with the molar ratio of monomers and the porogen type. With the use of chlorobenzene, their specific surface increased (from 357 to 500 m^2^/g) along with an increasing TMPSM content in the polymer matrix. This phenomenon is particularly valuable because it allows for the introduction of a high number of functional groups without losing porosity. A similar trend was reported for divinylbenzene-*co*-triethoxyvinylsilane copolymers [53]. A quite opposite tendency (deterioration of the surface area with an increasing amount of functional monomer) was observed for other monomers like glycidyl methacrylate hydroxyethylmetacrylate and *N*-vinyl-2-pyrrolidone.

Also, thermal degradation was strongly dependent on the copolymer composition. Namely, the higher the content of TMSPM, the higher the thermal stability in both inert and oxidative atmospheres. In helium, the thermal stability increased from 269 °C to 283 °C, while in synthetic air it increased from 266 °C to 298 °C, considering a 5% mass loss temperature. It is worth mentioning that TMSPM-*co*-TRIM_3 and TMSPM-*co*-TRIM_4 copolymers were more stable than pure poly(TRIM). The conducted investigation also showed that the thermal degradation process occurred through three stages and that the copolymers did not degrade completely. The final residues at 700 °C were in the range of 12–31% (in helium) and 17–26% (in synthetic air) of the starting masses. Analogously, like in the case of a porous structure, increasing the amount of the methoxysilane monomer contributed to an improvement of the multifunctional properties of the final copolymers.

It can further be stated that the newly obtained porous microspheres can be used as effective sorbents in many separation techniques, including high-temperature processes. GC analyses conducted at 200 °C show better resolution compared with analyses at 140 °C.

## Figures and Tables

**Table 1:** Denomination of copolymers and synthesis parameters.

Copolymer	Monomers Ratio	Diluents (mL)	
		Toluene	Chlorobenzene
TMSPM-*co*-TRIM_1T	1:1	22.5	-
TMSPM-*co*-TRIM_2T	2:1	22.5	-
TMSPM-*co*-TRIM_3T	3:1	22.5	-
TMSPM-*co*-TRIM_4T	4:1	22.5	-
TMSPM-*co*-TRIM_1C	1:1	-	22.5
TMSPM-*co*-TRIM_2C	2:1	-	22.5
TMSPM-*co*-TRIM_3C	3:1	-	22.5
TMSPM-*co*-TRIM_4C	4:1	-	22.5

**Table 2:** The statistical data of particle size distribution.

Copolymeric Microsphere	D(0.1)	D(0.5)	D(0.9)	Span
TMSPM-*co*-TRIM_1T	51	89	121	0.786
TMSPM-*co*-TRIM_2T	36	75	104	0.907
TMSPM-*co*-TRIM_3T	38	78	98	0.769
TMSPM-*co*-TRIM_4T	49	84	112	0.750
TMSPM-*co*-TRIM_1C	86	155	181	0.613
TMSPM-*co*-TRIM_2C	76	120	148	0.600
TMSPM-*co*-TRIM_3C	83	131	152	0.527
TMSPM-*co*-TRIM_4C	81	154	168	0.565

**Table 3:** The determined parameters of the porous structure of the investigated copolymers.

Copolymeric Microsphere	Specific Surface Area *S*_BET_ (m^2^/g)	Pore Volume *V* (cm^3^/g)	Pore Diameter *D*_BJH_ (Å)
TMSPM-*co*-TRIM_1T	457	0.325	3.8
TMSPM-*co*-TRIM_2T	432	0.176	3.8
TMSPM-*co*-TRIM_3T	422	0.132	3.8
TMSPM-*co*-TRIM_4T	382	0.086	3.4
TMSPM-*co*-TRIM_1C	357	0.154	3.8
TMSPM-*co*-TRIM_2C	456	0.137	3.6
TMSPM-*co*-TRIM_3C	444	0.133	3.4
TMSPM-*co*-TRIM_4C	500	0.116	3.4

**Table 4:** The swellability coefficients of the investigated copolymers.

Copolymer	Swellability Coefficient *B* (%)		
	Methanol	Toluene	Acetone
TMSPM-*co*-TRIM_1T	21	17	19
TMSPM-*co*-TRIM_2T	20	16	18
TMSPM-*co*-TRIM_3T	20	17	19
TMSPM-*co*-TRIM_4T	22	20	20
TMSPM-*co*-TRIM_1C	28	23	27
TMSPM-*co*-TRIM_2C	34	28	31
TMSPM-*co*-TRIM_3C	37	29	32
TMSPM-*co*-TRIM_4C	41	32	39

**Table 5:** The characteristic temperatures based on TG and DTG data determined in helium.

Copolymeric Microsphere	*T*_5%_ (°C)	*T*_20%_ (°C)	*T*_50%_ (°C)	*T*_max1_ (°C)	Δ*m*_1_ (%)	*T*_max2_ (°C)	Δ*m*_2_ (%)	*T*_max3_ (°C)	Δ*m*_3_ (%)	Residue (%)
TMSPM-*co*-TRIM_1T	269	303	380	277	21	379	54	608	13	12
TMSPM-*co*-TRIM_2T	276	323	409	293	20	399	50	668	10	20
TMSPM-*co*-TRIM_3T	282	339	432	290	16	410	48	678	11	25
TMSPM-*co*-TRIM_4T	283	356	443	288	14	418	47	712	8	31

**Table 6:** The characteristic temperatures based on TG and DTG data determined in synthetic air.

Copolymeric Microsphere	*T*_5%_ (°C)	*T*_20%_ (°C)	*T*_50%_ (°C)	*T*_max1_ (°C)	Δ*m*_1_ (%)	*T*_max2_ (°C)	Δ*m*_2_ (%)	*T*_max3_ (°C)	Δ*m*_3_ (%)	Residue (%)
TMSPM-*co*-TRIM_1T	266	298	360	286	30	349	43	537	10	17
TMSPM-*co*-TRIM_2T	267	308	376	288	15	357	53	568	11	21
TMSPM-*co*-TRIM_3T	291	320	394	305	25	361	38	562	13	24
TMSPM-*co*-TRIM_4T	298	332	412	307	20	369	42	574	12	26

## Data Availability

The data presented in this study are available on request from the corresponding authors.
